# Assessing physiological response mechanisms and the role of psychosocial job resources in the physical activity health paradox: study protocol for the Flemish Employees’ Physical Activity (FEPA) study

**DOI:** 10.1186/s12889-019-6950-7

**Published:** 2019-06-15

**Authors:** Margo Ketels, Dirk De Bacquer, Tom Geens, Heidi Janssens, Mette Korshøj, Andreas Holtermann, Els Clays

**Affiliations:** 1 Department of Public Health and Primary Care, University Hospital Ghent, Ghent University, entrance 42 (4K3), Corneel Heymanslaan 10, 9000 Ghent, Belgium; 2 Liantis, Occupational Health Services, Bruges, Belgium; 3 National Research Centre for the Working Environment, Copenhagen, Denmark

**Keywords:** Physical activity, Psychosocial, Occupational health, Work environment, Cardiovascular, Cardiac autonomic regulation, Heart rate monitoring, Accelerometers, FEPA

## Abstract

**Background:**

In the current labour system many workers are still exposed to heavy physical demands during their job. In contrast to leisure time physical activity (LTPA), occupational physical activity (OPA) is associated with an increased risk of cardiovascular diseases and all-cause mortality, termed “the physical activity (PA) health paradox”. In order to gain more insight into the PA health paradox, an exploration of structural preventive measures at the workplace is needed and therefore objective field measurements are highly recommended. The objective of this paper is to provide an overview of the protocol of the Flemish Employees’ Physical Activity (FEPA) study, including objective measurements of PA, heart rate (HR) and cardiorespiratory fitness (CRF) to gain more insight into the PA health paradox.

**Methods:**

A total of 401 workers participated in the FEPA study across seven companies in the service and production sector in Belgium. The participants comprised 167 men and 234 women, aged 20 to 65 years. OPA and LTPA were assessed by two Axivity AX3 accelerometers on the thigh and upper back. Ambulatory HR was measured by the Faros eMotion 90° monitor. Both devices were worn during two to four consecutive working days. In addition, CRF was estimated by using the Harvard Step Test. Statistical analyses will be performed using Pearson correlation, and multiple regression adjusted for possible confounders.

**Discussion:**

This study aims to provide a better insight in the PA health paradox and the possible buffering factors by using valid and objective measurements of PA and HR (both during LTPA and OPA) over multiple working days. The results of the study can contribute to the prevention of cardiovascular disease by providing tailored recommendations for participants with high levels of OPA and by disseminating the results and recommendations to workplaces, policy makers and occupational health practitioners.

## Background

Among many elements contributing to good health, leisure time physical activity (LTPA) is widely acknowledged as a major factor with a particularly beneficial role in the prevention of cardiovascular disease (CVD) [1–3]. The term “LTPA” refers to all types of physical activities, e.g. sports, recreation and transportation, which are not work-related and are performed outside the job setting. Given these beneficial effects, international guidelines advise at least 30 min of moderate to intense physical activity (PA) on at least five days a week [4].

On the other hand, various studies have demonstrated strikingly different health effects of PA that is work-related, i.e. occupational physical activity (OPA). Increasing evidence suggests that workers who regularly perform demanding OPA, show an increased risk for CVD [1, 2, 5], mortality [6] and long-term sickness absence [7]. These negative health effects are more pronounced among workers with low cardiorespiratory fitness [8] and low psychosocial resources [9]. The opposite effect of OPA and LTPA on various health parameters is known in the literature as “the PA health paradox” [10].

A second, somewhat related paradox pertains to the effect of cardiorespiratory fitness (CRF). On the one hand low CRF is a strong independent predictor of all-cause mortality and CVD [11, 12]. Increasing the level of CRF by means of exercise is therefore often seen as a possible solution to reduce the risk of CVD [13, 14]. This relation is probably due to a decreased strain from LTPA on the cardiovascular system [15, 16]. On the other hand caution is needed, since performing high LTPA in addition to high OPA in order to elevate the level of cardiorespiratory fitness might lead to a higher risk of developing CVD [17, 18]. The relation between OPA and CRF is furthermore unclear in the literature, which is characterized by many inconsistent findings [1, 6, 17].

A possible explanation for the different effects of OPA and LTPA on health and CRF can be found in the differences in intensity and duration of the activity [19]. OPA is generally characterized by prolonged exposure to static or anaerobic PA during many hours a day with limited opportunities to take breaks. The contents and temporal structure of LTPA on the other hand can be adjusted to the individual’s needs and preferences. LTPA is furthermore characterized by dynamic movements of large muscle groups, which induces a cardiovascular training effect, whereas OPA can cause cardiovascular overload due to increased stress on the arterial wall, in turn causing atherosclerosis [20] or sustained elevated blood pressure [21].

Given the considerable amount of workers with high levels of OPA in Europe [6, 20, 22] and the increasing evidence of the harmful effects of OPA on cardiovascular disease, it is necessary to develop primary preventive measures against premature cardiovascular morbidity and mortality among workers with high levels of OPA.

A beneficent psychosocial work environment [9] could counterbalance the detrimental effect of OPA on cardiovascular disease. According to the Job-Demand-Control-Support model [23, 24], job demands, i.e. workload and time pressure as well as physical and emotional demands, can have harmful effects on workers. These effects can however be moderated by the buffering factors of job control and social support [9, 25, 26]. The buffer hypothesis states more in particular that job control may prevent job demands from increasing the risk of CVD. Moreover, the combination of high demands and low control, which is named high strain and is supposed to be the most adverse health effect, can be moderated by social support [27]. The counterbalancing effects of psychosocial resources can operate both in an indirect and a direct way. Whereas the Job-Demand-Control-Support model focuses on the indirect ways job control and social support buffer the harmful effect of OPA on CVD, a more direct way for job resources to lower the cardiovascular reactivity in response to physical work load has been proposed as well [26].

Detailed objective physiological assessments of the cardiac effects are needed to shed some light on the mechanical counterparts of the buffering resources as well as on the underlying mechanisms of the PA health paradox in general. Ambulatory measurements of heart rate (HR) and heart rate variability (HRV) are particularly relevant in this context. Ambulatory HR throughout the day showed to be an independent predictor for all-cause mortality [18], while HRV is considered as a reliable indicator of cardiac autonomic regulation [28]. In occupational health research, reduced HRV has been associated with work stress and is often used as a physiological marker of work-related musculoskeletal disorders [29, 30]. Reduced HRV furthermore predicts CVD as well as all-cause mortality [31–33]. By using objective measurements of PA and HR a number of recent studies gained more insight into the underlying mechanisms of the PA health paradox, focusing on intensity of OPA [34], mean HR and HRV [35].

While the aforementioned studies certainly have added to our understanding of the relation between OPA and health, further research based on objective measurements will be needed to gain a better understanding of all factors involved and complement the existing literature. Only few studies mainly based on Danish working populations are available [34–36]. Moreover, evidence about the buffering effect of psychosocial job resources in the relation between OPA and the risk of CVD is scarce so far.

## Methods

### Aims

The overall aim of this paper is to describe the protocol of the Flemish Employees’ Physical Activity (FEPA) study. The objectives of the FEPA study are (a) to examine physiological responses associated with detailed objective assessments of different types of LTPA and OPA activities and (b) to examine the moderating (i.e. buffering) impact of psychosocial job resources on the cardiac autonomic regulation connected to OPA.

### Study population and design

**Fig. 1:**
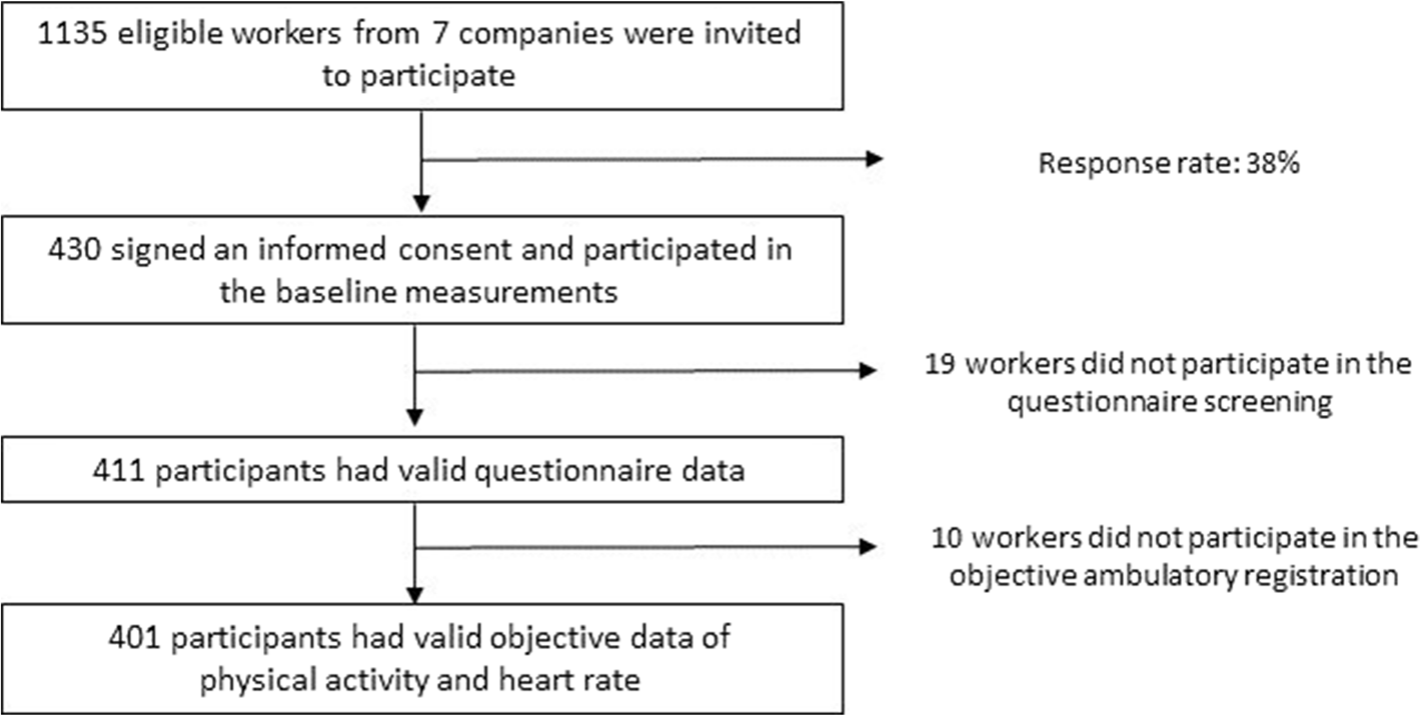
Flowchart of the recruitment of the study population

The FEPA study was a cross-sectional study that recruited participants by convenience sampling across different companies in Flanders (Northern region in Belgium). The companies were located in the manufacturing and service sector, where most employees had  considerable levels of OPA. The inclusion criterion at workplace level was the approval of the companies for data collection to take place at the workplace during paid working hours. A total of 1135 eligible workers from 7 companies were contacted and invited to participate voluntarily in the study. A total of 430 workers were willing to participate in the study and signed an informed consent. All workers met the following inclusion criteria: non-pregnant, a sufficient knowledge of the Dutch language, employment rate of at least 50%, and no exclusive nightshift worker. From February 2017 until June 2018, the 430 workers were enrolled in the study, corresponding to an initial participation rate of 38%. Eventually, 401 participants had complete valid data. The sample included 167 men and 234 women, aged between 20 and 65 years old, with a mean age of 39.15 (± 11.04 year), and consisted of 19.2% (= 77 participants) administrative workers. A more detailed overview of the flow of the recruitment of the study population is shown in Fig. 1.

The study was approved by the Research Ethical Committee of Ghent University Hospital (number 2017/0129). After the study had taken place, participants received an individual health-related feedback report, serving as an incentive for participation. The companies received a general feedback report regarding physical and psychosocial risk exposures at group level.

### Procedure

The test protocol included a self-reported questionnaire, a baseline medical screening and objective ambulatory measurements of PA and HR. After receiving a study invitation letter, participants that showed interest were provided an appointment for the baseline screening, together with the questionnaire. Participants were requested to fill in either the questionnaire on paper and bring it to the screening that took place at the worksite during working hours or to fill in the questionnaire online. The participants received information about the objectives and procedure of the study and were requested to give an informed consent. A trained researcher conducted baseline measurements including height, weight, waist circumference, blood pressure and estimation of the CRF. After the tests, the researcher attached two accelerometers and a HR monitor on the skin of the participants for objective measurements of respectively PA and HR. The participants were asked to wear the devices continuously for three to four consecutive working days, 24 h a day, which resulted in a mean of 3.05 (± 0.88) wear days. Participants were asked to keep a diary during the days of recording to describe their day scheme. After the period of objective measurements, the participants returned their equipment and diary to the researcher.

### Sample size calculation

An a priori sample size of minimum 360 participants was targeted. For addressing the research questions, a number of multiple linear regression models will be conducted assessing OPA and LTPA as main predictors in relation to HR and HRV outcome parameters. These models should allow for controlling the effect of main confounders – in particular sex, age, educational level, body mass index (BMI), smoking and CRF – and for including interaction terms with psychosocial variables. A sample size calculation showed that the minimum required sample for a multiple regression study including 10 predictors with a medium anticipated effect size was 118, given a desired statistical power level of 80% and a probability level of 5% [37]. However, for accurately estimating population parameters in observational studies, it has been recommended that the ideal sample size should preferably be at least 300 [38]. We further accounted for an anticipated drop-out of 10%, and missing data of 10%, resulting in a final targeted sample of 360 participants.

### Data collection

#### Questionnaire data

The first part of the questionnaire, i.e. the socio-demographic part, contained general questions about age (years), sex (male of female), marital status, education level and nationality (Belgian or other nationality). For education level, until primary school was classified as a low educational level, secondary school and/or 1 to 2 years of specialization as a medium educational level, and high school or university as a high educational level.

In the next part of the questionnaire, participants were asked information about their occupation. All workers reported their work features, such as their specific job type, seniority, work schedule and working hours per week. Furthermore, the Occupational Sitting and Physical Activity Questionnaire (OSPAQ) was used to subjectively assess occupational sedentary behaviour and PA. The OSPAQ has the advantage of allowing a comprehensive analysis of time spent on various types of activities, i.e. sitting, standing, walking and physically demanding work [39, 40]. The following question was used to assess the physical workload: “On a scale of 0-10, how physically demanding is your job on a regular working day? (0= not at all demanding, 10= very demanding)”. Furthermore, seven questions [41, 42] on a 6-point Likert scale rated from “never” to “almost always” were used to capture the amount of lifting, carrying, extreme heat or cold, very loud sounds and whole body vibrations during working hours. This method was used to overcome the problem raised by the impossibility to measure this objectively with the accelerometers.

A detailed assessment of psychosocial job resources was conducted, mainly based on the insights provided by the theoretical framework of the Job-Demand-Control-Support [43] model. More specific, the Job Content Questionnaire (JCQ) was used to chart the psychosocial characteristics of the jobs involved [43]. Questions regarding job demands (five items), job control (nine items) and social support (eight items) were rated by the participant on a 4-point Likert scale ranging from “totally disagree” to “totally agree”. The final score on job demands was calculated by taking the mean of the scores on the five questions pertaining to job demands. The final score for job control or decision latitude was calculated by taking the mean of the sum of two subdimensions that are highly correlated: skill discretion, i.e. the level of skill and creativity required on the job (6 items), and decision authority, i.e. the possibilities for workers to take decisions about their work (3 items). The final score of social support at the workplace was also calculated by taking the mean of the sum of two subscales, i.e. supervisor support (4 items) and co-worker support (4 items). The validity and reliability of the Job Content Questionnaire to measure psychosocial work situations and job strain among various occupations has been confirmed in many studies [44, 45].

Furthermore, the JCQ contained specific measures of physical exertion (high physical effort, lifting heavy loads, rapid physical activity), two items assessing isometric loads in awkward body positions, and awkward positions above head or arms. The items were scored on a 4-point Likert scale ranging from “totally disagree” to “totally agree”. Additionally, the subjective perception of current workability was evaluated using a question “How many points would you give to your current workability?”. The Work Ability Score, abbreviated as WAS [46], was obtained by the workers’ answer on this question by rating a 10-point Likert scale, that ranged from “not capable to work” to “best workability”. A high score on this scale represents a high subjective perception of current workability.

Some additional instruments regarding social capital (i.e. Finnish Public Sector Study) [47], need for recovery (i.e. 11-item NFR scale) [48], work engagement (i.e. UBES) [49] and work-family conflict [50] were administered as well.

The last part of the questionnaire consisted of questions regarding health and well-being, e.g. length (centimetres), weight (kilograms), smoking status, the amount of alcoholic drinks, coffees and caffeine containing drinks consumed per week or day.

Furthermore, the physical activity scale (PAS2) [51] was used to measure the PA during work, transportation and leisure time. This questionnaire consists of nine items, including four items measuring time spent on different daily activities and three items measuring time spent on weekly activities. The subpart measuring daily PA contains questions about duration of sleep per weekday, sedentary behaviour and OPA, leisure time and the time commuting to and from work. On a weekly basis, the questions inform about light (e.g. walking, light cleaning…), moderate (e.g. gardening, moderate strenuous sports such as swimming, bicycling…) and vigorous (e.g. running, soccer…) PA during leisure time.

Further questions about the amount of fruit, vegetables, snacks and fast food consumed were answered by means of a 4-point Likert scale (i.e. never, 1–2 times a week, 3–4 times a week or daily). Subsequently, the participants indicated whether they suffered from a certain condition in order to capture their medical history (i.e. heart disease, high blood pressure, high cholesterol, respiratory disease, mental illness, cancer, diabetes or other). One item asked about the perceived cardiorespiratory fitness in comparison with peers of the same sex and age [52]. Possible answers were “worse than my peers”, “equal to my peers” and “better than my peers”.

The General Health Questionnaire (GHQ) [53] was used to capture information about the mental health of participants and is a tool to identify common psychopathological conditions. The items in the questionnaire were rated on a 4-point Likert scale (from "less than usual" to "much more than usual"), whereby 6 items are negatively scored and 6 are positively scored. The short questionnaire (including 12 questions) is a valid and reliable tool for all ages from adolescent upwards [54].

Self-reported information about low back pain and neck and shoulder pain was obtained using a modified version of the Standardized NORDIC questionnaire for the analysis of musculoskeletal symptoms [55]. A first general question asked if the participants have had any low back pain in the last 12 months. If the answer was positive, the participants had to specify the specific duration in days and had to answer if they had low back pain specifically during the last month. A set of similar questions was included pertaining to neck and shoulder pain.

The last questions of the questionnaire asked about health literacy (i.e. Health Literacy Survey-EU-Q6) [56] and positive and negative affectivity (i.e. Positive and Negative Affect Schedule, PANAS) [57].

#### Baseline medical examination

##### Resting blood pressure and heart rate

The blood pressure and HR were measured on the right arm after the participants had already been sitting down for 10 min. Measurements of the blood pressure and HR were done twice with the OMRON M6/M6 AC (OMRON Corporation, Kyoto, Japan), with a five minutes pause in between. The average of both scores was taken as the final score for respectively blood pressure and HR at rest.

##### Anthropometrics

The length (meter) and weight (kilogram) were measured with a Seca 704 column scale (SECA Medical Measuring Systems and Scales, Birmingham, UK; scales 701/704). Before standing on the platform of the scale the workers were asked to remove heavy outer garments (jackets, heavy sweaters and others, belts, watches etc.) and shoes. Height was measured while the participant was standing straight forward and wearing no shoes. Based on the outcome, the corresponding BMI (kg/m^2^) was calculated as body weight (kg) divided by the square of height (m). In line with international standards defined by the World Health Organization, overweight was defined as a BMI of 25 kg/m^2^ or higher; those with a BMI of 30 kg/m^2^ or higher were classified as obese. The waist circumference was measured with a tapeline that expands less than others since it was made out of plastic. The waist was defined as the narrowest point between the lowest rib and the iliac crest.

##### Harvard step test

**Fig. 2:**
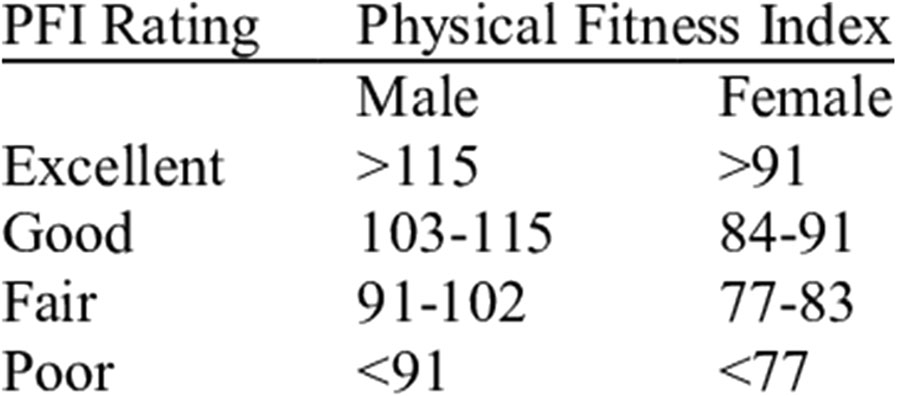
Physical Fitness Index rating (Fox et al., 1973)

The Harvard step test (HST) [58] is a single-stage test used to determine the physical fitness index (PFI). The workers were required to step up and down on a bench of 33- or 40 cm high, for respectively women and men. Participants were allowed to use the same foot continuously as the first foot to perform the exercise. It is however advisable to change the foot that is used to step up the bench once every minute. When stepping up the bench the knee needed to be completely extended. Jumping was prohibited. Before the actual test was conducted, participants received information about the testing procedure and they had an one-minute practice moment to become acquainted with the test protocol. The participants had to follow a stepping rate of 22.5 steps per minute during a period of 5 min, set by a metronome. Stopping before the end of 5 min can be due to two reasons, either the participant had to stop due to exhaustion, or the stepping rate of 22.5 steps was not maintained for longer than 15 s. After completing the test, or after exhaustion, the participants were asked to take a seat and three recovery heart rates (in beats per minute; bpm) were measured with a polar device after 1, 2 and 3 min (using a stopwatch), respectively heart rate 1, 2 and 3. The PFI was determined by the following equation: PFI % [59] = (Duration of exercise in seconds × 100)/(2 x (heart rate 1 + 2 + 3)). For example, if the total test time was 300 s (if completed the whole 5 min), and the three recovery heart beats were respectively 100, 90 and 80, the physical fitness score would be: (300 × 100)/(2 × 270) = 55.55. The physical Fitness Index rating is shown in Fig. 2.

#### Objective ambulatory registration

The objective ambulatory registration consisted of two main measurements. First, the participants were asked to wear two accelerometers (Axivity AX3) for measuring the PA. Second, participants were asked to wear a HR monitor (Faros eMotion 90°) to collect interbeat intervals (IBIs). The accelerometers and HR monitor were worn at the same time and for three to four consecutive working days, 24 h a day. The participants were instructed to wear the equipment during the whole measurement period, excepted for the HR monitor that was not water resistant which means that the device must be removed before taking a shower, bath or any kind of activities whereby the participants’ body came into contact with water. The participants were also instructed to remove the equipment if it caused any kind of discomfort. Furthermore, a paper-based diary was used by the participants to note their work time, leisure time, sleep and non-wear hours, as well as time of the reference measurements. A reference measurement means that the participants had to stand still in an upright and neutral position for 15 s each day. The exact time and date of the reference measurement had to be noted in their diary. This reference measurement is necessary to obtain the coordination between the axis of the accelerometers and the orientation of the thigh and trunk.

##### Accelerometers

One accelerometer (Axivity AX3) [60] was placed in the middle of the back (at the level of T1-T2 on the processus spinosus) and the other one in the middle of the right thigh (at the front of the thigh and midway between the iliac crest and the upper border of the patella). The accelerometers were orientated with the x-axis and the USB port pointing downwards, y-axis horizontally to the left and z-axis horizontally forward. The accelerometers were placed on the skin by using Opsite Flexifit wound foil. The device is waterproof and therefore suitable for constant use.

##### Heart rate monitor

Electrocardiography was measured by using a Faros eMotion 90° device (Bittium) [61], a compact HR monitor that can be worn day and night, excepted during activities where the device would be in contact with water. After every switch-off period the time-period had to be noted in the diary of the participants. The small device (10 g, 18.8 cm by length) was connected with two pre-gelled silver chloride (Ag/AgCl) electrodes, Ambu BlueSensor longterm (VL-00-S/25) by a short lead. After cleaning the skin with ethanol solution, the first electrode was placed under the right collarbone and connected with a second electrode that was placed above the left hip.

### Data processing and statistical analysis

#### Accelerometer data

Acceleration data were sampled in three dimensions with a dynamic range of approximately 8 G (1 G = 9.81 m/s^2^), a precision of 12 bit and a frequency of 25 Hz. Initialization for recording, synchronization between the two accelerometers, and downloading the data afterwards was executed by using software from Axivity (AX3-GUI, Omgui software). For the analysis of the data, a custom-made MATLAB based software named Acti4 was used (The National Research Centre for the Working Environment, Copenhagen, Denmark and Federal Institute for Occupational Safety and Health, Berlin, Germany). This program is capable of determining the type and duration of different activities (e.g. lying, walking, running, sitting, standing, walking on stairs, rowing and cycling) as well as the amount of steps with high sensitivity and specificity [62]. More specifically, the accelerometer at the thigh identified sitting, standing, walking, walking on stairs and running. Activities were classified as static if the value was lower than 0.1 G. Values between 0.1 G and 0.72 G were classified as walking and above 0.72 G the activity was classified as running. Furthermore, walking on stairs was identified by using the forward/backwards angle of the thigh. Static activities such as sitting were determined with an inclination of the longitudinal axis of the thigh accelerometer above 45°, and standing for values less than 45°. The difference between lying and sitting can be established by using the accelerometer on the back whereby lying is identified as an inclination of the x axis above 65°. Last, the activity type ‘moving’ is a term that refers to a standing position with small movements but without regular walking [62]. To establish the difference between standing and moving, the standard deviation of the maximum acceleration in all the three the axis’s was used. Values that were below 0.1 G were classified as standing and values above 0.1 G as moving.

Furthermore, all the accelerometer data were manually divided in the Acti4 software into intervals with regard to the information found in the diaries (i.e. before working hours, during working hours, after working hours and sleep after working hours). Periods were identified as non-wear time if (a) the Acti4 software detected a period longer than 90 min of no movement, (b) the participant reported non-wear time on their diary, or (c) artefacts or missing data were detected by the Acti4 software. The data for a workday were considered to be valid if there were objective measurements of at least 4 h of working time for that day or measurements of 75% of the individual’s average reported working time. The data for a leisure time day were included if there were objective measurements of at least 4 h of leisure time or measurements of 75% of the individual’s average reported leisure time. The data on a daily basis were only included in the data pool if a minimum of 10 h of data was available. These cut-off values were implemented to prevent bias due to inclusion of extreme unrepresentative data and to reflect optimal daily wear time.

#### Heart rate and heart rate variability data

The Faros eMotion 90° measures raw signals with a sensitivity of 0.25 mV, a dynamic range of approximately 4 G and is able to estimate the HR and HRV. HR is calculated by taking into account the stored interbeat intervals (IBIs) between the R peaks in the QRS complex. The IBIs were downloaded by the eMotion EDF viewer, a software application that can only show the data. Further analysis was therefore executed by Kubios software [63]. IBIs were considered erroneous and were discarded from further analysis if they corresponded to a HR less than 36 or more than 200 bpm, or if deviated more than 15% compared to the neighbouring IBIs. Moreover, measurement periods with a resulting error rate above 50% were discarded. For the rest of the measurements periods, the interbeat series were resampled with a frequency of 4 Hz using a linear interpolation scheme. The resampled and interpolated series were then used for the calculation of HR values. If the device measured at least 4 h of working time and a total of more than 7 h during all recorded working periods (work and leisure time activities combined), it was considered to generate representative data.

To calculate the HRV parameters the software program R 3.5.1 and the package RHRV was used [64]. The output, consisting of Kubios Text files, was added into R. A selection of HRV parameters (see Table 1) was calculated by following the procedure described in “Heart Rate Variability Analysis with the R package RHRV”.

**Table 1:** Overview of the HRV parameters

Parameter	Unit	Description
Time-domain measures		
Mean HR	bpm	Mean heart rate (reflects physical activity and sympatho-vagal balance)
Mean RR	ms	Mean of the selected beat-to-beat RR interval series (inversely proportional to mean HR)
SDNN	ms	Standard deviation of the IBI of normal sinus beats (demonstrates overall HRV)
HRVindex	ms	Integral of the density of the RR interval histogram divided by its height
SDANN	ms	Standard deviation of the averages NN (normal-to-normal) intervals for each of the 5 min segments during a 24 h recording
RMSSD	ms	Root mean square of successive RR interval differences (reflects mainly parasympathetic activation of the autonomic nervous system)
pNN50	%	Percentage of successive NN intervals that differ by more than 50 ms (NN50) divided by the total number of all NN intervals
Frequency-domain measures		
HRV	ms^2^	Total energy across all spectral bands of the frequency analysis
LF power	ms^2^	Absolute power of the low-frequency band (frequency range 0.04–0.15 Hz) (reflects both sympathetic and parasympathetic activation)
HF power	ms^2^	Absolute power of the high-frequency band (frequency range 0.15–0.4 Hz) (reflects parasympathetic activation)
LF/HF	–	LF/HF power ratio (estimates sympatho-vagal balance)
Non-linear measures		
SD1	ms	Poincaré plot standard deviation perpendicular the line of identity
SD2	ms	Poincaré plot standard deviation along the line of identity

*Abbreviations: HR* heart rate, *bpm* beats per minute, *RR* interbeat, *IBI* interbeat interval, *HRV* heart rate variability, *NN* normal-to-normal, *Hz* Hertz, *LF* low frequency, *HF* high frequency, *SD* standard deviation

#### Statistical analysis

Before conducting further analyses, the distribution of all parameters will be checked and boxplots will be applied to detect outliers. Distribution of normality of continuous variables will be examined using the Shapiro-Wilk test. Descriptive statistics of baseline characteristics will be reported through numbers and proportions. T-test and chi-square tests will be used in order to compare the characteristics. Pearson or Spearman correlations and multiple regression analyses will be used to analyse the data, adjusting for possible confounding variables (sex, age, educational level, smoking, BMI and CRF). All analyses will be conducted using SPSS software (version 25.0, SPSS Inc., Chicago, Illinois) and the level of significance will be set at *p* < 0.05 (5%).

## Discussion

The overall aim of this paper was to describe the protocol of the FEPA study and to contribute to the debate regarding the PA health paradox by describing in detail possible methods that can be used to measure both the moderators and some possible underlying mechanisms. A better insight in the PA health paradox and the possible buffering factors can contribute to the prevention of cardiovascular disease by tailored recommendations for participants with high OPA.

### Strengths

The FEPA study has several specific strengths compared to a number of previous studies. The main strength of this study is the use of objective ambulatory measurements, which decrease the risk of self-reported bias. In previous studies, self-reported instruments were used to capture PA in both working and leisure time activities [1]. These instruments are generally known to have limited reliability and validity [65, 66] and are also possibly subjected to recall bias. Furthermore, self-reported instruments are not able to simultaneously capture all dimensions and temporal activity patterns of PA [67]. The objective monitoring methods in the study are therefore essential to avoid these limitations and to provide valid measurements of different PA types over multiple days, which improves the repetitiveness of the measurements [68].

Another major strength of the study is the inclusion of CRF data. Evidence suggests that the level of fitness may be a confounder in the interplay between LTPA and OPA on CVD [8, 69]. Another strength is the relatively large sample size including both men and women. Studies showed that men and women may be differently exposed to physical and psychosocial risk factors at work [70], which emphasizes the need of a sample including both men and women to contribute to the generalizability of findings.

### Limitations

Besides the main strengths of the study there are also some potential limitations that need to be taken into account. The first limitation is that physical workload and leisure time PA were only measured on working days, which prevent us from drawing conclusions from non-working days, and thus to total LTPA.

Since our sample was selected by using convenience sampling, it may not be considered to be representative of the general population. Additionally, participation to the study was voluntary, which can lead to participation bias. The problem arises in particular since it is unclear whether this study aroused more interest in the younger and more fit workers. A second, and related limitation is the recruitment strategy through workplaces and the necessity of conducting all measurements during working hours, which may lead to selection bias where only the companies with higher resources may choose to participate. Finally, due to the cross-sectional design of the study, it will not be possible to assess causal relations. Given these potential limitations, prospective studies are to be recommended in future research.

## Conclusion

This study, based on technical measurements, will contribute to the understanding of the potential underlying mechanisms and the possible psychosocial buffering resources of the PA health paradox. Physically demanding tasks are still and will continue to be an important reality for a large number of people in the work environment. From a public health perspective, knowledge about prevention strategies and more specific the implementation of the strategies are needed to provide a better environment for workers with high physical demands to perform their jobs in a safe way, thus avoiding cardiovascular overload.

## Abbreviations

BMI: Body mass index
bpm: beats per minute
CRF: Cardiorespiratory fitness
CVD: Cardiovascular disease
FEPA: Flemish employees’ physical activity
GHQ: General health questionnaire
HF: High frequency
HR: Heart rate
HRV: Heart rate variability
HST: Harvard step test
Hz: Hertz
IBIs: Interbeat intervals
JCQ: Job content questionnaire
LF: Low frequency
LTPA: Leisure time physical activity
NFR: Need for recovery
NN: Normal-to-normal
OPA: Occupational physical activity
OSPAQ: Occupational sitting and physical activity questionnaire
PA: Physical activity
PAS: Physical activity scale
PFI: Physical fitness index
RR: interbeat
SD: Standard deviation
WAS: Work ability score
